# Effects of endocardial microwave energy ablation

**Published:** 2005-07-01

**Authors:** Vicente Climent, Aquilino Hurlé, Siew Yen Ho, Damián Sánchez-Quintana

**Affiliations:** * Departamento de Anatomía Humana, Facultad de Medicina, Universidad de Extremadura, Badajoz, Spain;; † Departamento de Cirugia Cardiaca del Hospital General Universitario de Alicante, Alicante, Spain;; ‡ National Heart and Lung Institute, Imperial College, and Royal Brompton and Harefield Hospitals, London, UK;

**Keywords:** atrial fibrillation, microwave ablation

## Abstract

Until recently the treatment of atrial fibrillation (AF) consisted primarily of palliation, mostly in the form of pharmacological intervention. However because of recent advances in nonpharmacologic therapies, the current expectation of patients and referring physicians is that AF will be cured, rather than palliated. In recent years there has been a rapid expansion in the availability and variety of energy sources and devices for ablation. One of these energies, microwave, has been applied clinically only in the last few years, and may be a promising technique that is potentially capable of treating a wide range of ventricular and supraventricular arrhythmias. The purpose of this study was to review microwave energy ablation in surgical treatment of AF with special interest in histology and ultrastructure of lesions produced by this endocardial ablation procedure.

## Introduction

Atrial fibrillation is known to be the commonest cardiac rhythm disturbance and a significant source of associated mortality and morbidity [1-3]. Its medical treatment with antiarrhythmic drugs has proved rather disappointing, with reported failure rates for maintaining sinus rhythm exceeding 80% in the medium to long term. Thus, the direct consequence of this poor therapeutic efficacy of antiarrhythmic drugs to restore sinus rhythm is to limit pharmacological treatment of chronic AF to the prevention of thromboembolic events and heart rate control in most instances.

## Surgical Procedures in AF treatment

In recent years, efforts have been made to develop more efficient surgical options to treat AF. During the decade of the 1980’s, Cox and colleagues [4-6] conducted some pioneering electrophysiologic research that lead to the development of the so-called Maze procedure [7], a surgical technique (modified in three occasions) that basically consisted of performing a set of transmural surgical incisions at precise locations of both atria in such a way that the resulting fibrous scars would produce lines of electrical isolation, so interrupting all possible re-entrant circuits necessary to perpetuate AF.

The outstanding results obtained with this technique [8,9], with reported success rates above 90% for curing the arrhythmia, justified its adoption as the gold standard for the surgical treatment of AF. However, this “cut and sew” Maze procedure did not gain great popularity among surgeons as it is complex, time consuming and not without potentially dangerous associated complications. So, further clinical and technological research was necessary in order to develop simpler, quicker and safer surgical techniques to deal with AF.

The complex conventional Maze III procedure [10] included left atrial lesions encircling all four pulmonary veins, extending towards the mitral annulus (the so-called left atrial isthmus) and towards the left atrial appendage, which was excised. Also, several right sided lesions, along the crista terminalis with extensions towards interatrial septum and also towards the free atrial wall and tricuspid annulus and excision of the right atrial, were described for this procedure.

However, the debate is now open on whether all atrial lesions described for the conventional Maze procedure are necessary in order to achieve optimal results in terms of reversion of AF into sinus rhythm. The relevant studies carried out by Haissaguerre et al [11] demonstrated that paroxysmal (intermittent) AF is induced by triggers located in the pulmonary veins in about 90% of cases. Consequently, the great majority of patients with paroxysmal AF could be cured by just electrically isolating the pulmonary veins, thus, simplifying considerably the lesion pattern described for the Maze procedure.

A different approach should be made regarding chronic (permanent) AF. The drivers that sustain chronic AF are also a matter of debate. Some authors [12,13] support the idea that AF drivers are located, as are the triggers, within the pulmonary veins and, on this basis, simple pulmonary vein isolation should be enough to treat chronic AF, as was the case with intermittent AF. Other authors [14] describe some macro-reentrant circuits on both atria, a phenomenon known as atrial electrical remodelling, as the drivers for permanent AF. If this was the case, pulmonary vein isolation alone would not be quite enough to cure the arrhythmia as the procedure would interrupt the triggers that initiate AF, but not the drivers that perpetuate it.

From the clinical point of view, the so-called partial or mini-Maze procedures, that is, procedures that include only left atrial lesions, have proved less effective than the Maze procedure itself, with reported success rates in the range of 60 to 80% [15]. Furthermore, postoperative atrial tachycardia secondary to re-entrant circuits around the mitral or tricuspid annuli can be expected in about 10% of patients treated with this limited approaches.

From all these data, we could conclude that: a) pulmonary vein isolation should be performed in all instances, b) paroxysmal AF requires further lesion lines in left atrial isthmus and cavo-tricuspid isthmus to prevent postoperative atrial re-entry tachycardia and c) permanent AF requires additional right-sided lesions to interrupt macro-reentry [16].

Another line of research on the surgical treatment of AF deals with the method to produce atrial lesions. Performing surgical incisions and subsequent suturing, as described for the conventional Maze procedure, guarantee transmurality but increase technical complexity and potential hazards. This is why, in recent years, several alternative forms of energy have been introduced with the aim of producing transmural atrial lesions that result in fibrous scars, similar to those described for the Maze procedure, but in a simpler, quicker and safer manner. These energy sources include radiofrequency, cryoenergy and microwaves, all of them available for clinical use, and others, such as laser energy or ultrasounds, which are at present being tested on experimental grounds. Recent data demonstrate that these energy sources are just as effective as surgical incisions to produce the desired atrial lesions [17-19] and considerably shortened the time taken for creating the maze in comparison to the Cox’s maze procedure. [20] Therefore, the conventional “cut and sew” technique has now been abandoned by most surgeons, even by Dr. Cox himself. Microwave and radiofrequency ablation yield similar results in terms of restoration of sinus rhythm following the Cox Maze III line concept, even in patients requiring complex double, or triple, valve procedures [21].

## Histology of the lesions produced by means of microwave energy

Microwave ablation is based on the effect of high frequency electromagnetic radiation on the atrial wall. These electromagnetic waves, when applied to the atrial endocardial or epicardial surface, interact with tissue dipolar molecules, mainly with water molecules, inducing their oscillation at a very high speed, so converting electromagnetic energy into kinetic energy. This high speed vibration favours friction between water molecules within the myocardial wall, which results in an increase of myocardial tissue heat [22]. Microwave ablation has been demonstrated effective for the treatment of atrial arrhythmias and because microwave antennas may be made into flexible linear applicators, they may be well suited to the ablation of AF and the production of catheter-based maze procedures. Thus, following these mechanisms, microwave energy has proved capable to produce histologically demonstrated transmural atrial lesions [23,24].

A search of the main medical journals was carried out to review the application of microwave on the epicardial or endocardial surfaces (Table 1). Some authors [9,25,29,31,32] prefer an epicardial approach which might allow reduction, or even elimination, of the aortic cross-clamp time and permits a surgeon to avoid an atriotomy when it not required for treating intracardiac pathology [23]. In general, epicardial ablation is safer than endocardial ablation because the energy source is directed into the atrial chamber rather than outward into adjacent mediastinal structures, and it also allows the operator to center the energy of application on the structures that contain more water and overcome fat tissue barriers. In spite of that, the endocardial application of microwaves is most widely used [26,30,33,34] (Table 1) in clinical series due to the great number of cases of chronic AF requiring surgical interventions on the mitral valve (repairs or replacements).

The electromagnetic fields can propagate through blood, desiccated tissue or scar and be deposited directly in the deeper tissue. The penetration depth depends upon several factors, including the dielectric properties of the tissue, frequency of the microwave energy, and antenna design [25,26] Several studies [27,28] have shown how the composition and thickness of the cardiac layers are major determinants in the formation of the lesion after ablation.

Similarly, Santiago et al [27] reporting on epicardial radiofrequency (RF) applications suggest that the thickness and the composition of the epicardium and the myocardium play an important role in the formation of the tissue lesion, and that this may account the large variability in the clinical results from different groups working in RF ablation. From a biophysical point of view, it is likely that atrial tissues with different compositions (more or less fat, connective and myocardial tissues, intercellular space, etc) will have different electrical properties. Fuller and Wood [28] in an experimental study using RF ablation suggested that the flow through an intramural coronary artery may play a role in preserving the myocardium around the vessel, and it is necessary to have a high tissue temperature to overcome the effect of intramural vascular cooling. They found the volume of preserved myocardium correlated directly with arterial flow rate through the lesion

Microwave energy has been used at frequencies of 0.915 GHz and 2.450 GHz for the myocardial ablation of arrhythmias (Table 1). Normally the energy is set around 65W in humans, and the application time is 45 seconds when applied endocardially on pulmonary veins. Other authors apply microwave energy epicardially for longer time (90 seconds) on the right atrial appendage (human), [23] or pulmonary veins (mongrel dogs) [29].

Keane et al [26] evaluated the effect of the microwave energy on a phantom tissue (which have similar properties to myocardium) and on ventricles of goats. It has been demonstrated (on the phantom tissue) that the antenna is capable of heating the tissue-equivalent material to at least 48°C and this was the temperature required for irreversible tissue damage and a depth of 8 mm. The desired result was achieved with a 2 minutes microwave exposure time at antenna power levels ranging from 22 to 34 watts (Table 1). Shorter exposure times of 30 and 60 seconds resulted in reduced penetration depth. At present, therefore, given the widespread microscopic injury by microwave ablation, additional data using different antennas and frequencies are needed to demonstrate the safety and efficacy of microwave.

Santiago and colleagues [27] reporting on tissue damage by heating induced by RF ablation in patients with mitral valve disease obtained similar lesions depth with 80-90°C in epicardial applications and 70°C in endocardial applications. The percentage of transmural lesions in this study was 8%, although according to other workers [23] it is not clear whether transmurality is a prerequisite for a lesion to be effective. The interruption of an electrical pathway may be effective although not all the fibres are destroyed. A thinner tissue might conduct slow enough to interrupt re-entrance phenomena and be sufficient to restore regular atrial activity.

**Figure 1:**
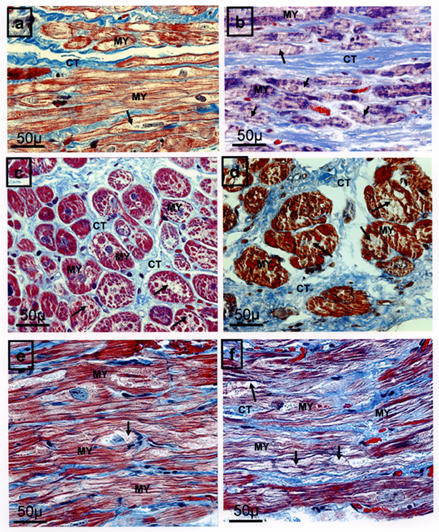
Histologic samples from the left atrial wall showing the myocardium prior to microwave ablation (**a,c,e**) compared with immediately after ablation (**b,d,f**). Note that hypertrophy, irregular shape, damage to myocytes (arrows), edema, and increased distance between cells are more evident in postablation samples. CT= Connective tissue, MY= Myocyte. The bar indicates 50μm

Most histological investigations of effects of microwave on tissues are made using light microscopy with stain techniques which distinguish clearly between extracellular and muscular tissue (Masson or Jones trichrome, elastic van Gieson, etc) and they compare samples of preablation with postablation or control tissues (Figure 1). As can be seen in Table 1, few data are available regarding the histologic acute-, short- and long-term changes of the myocardium after microwave hyperthermic exposure. The histologic examination of acute morphologic effects [24] after microwave endocardial ablation demonstrated that left atrial wall thickness appeared reduced when compared with preablation samples (Table 1). The samples after microwave ablation revealed a transmural lesion. Nevertheless, some apparently viable cells were observed near the epicardial side [24]. Atrial or ventricular myocytes in postablation showed severe myolysis, intramyocardial edema and intramural haemorrhage or foci of coagulative necrosis [23-26,29,34] (Table 1 and Figure 1).

**Figure 2:**
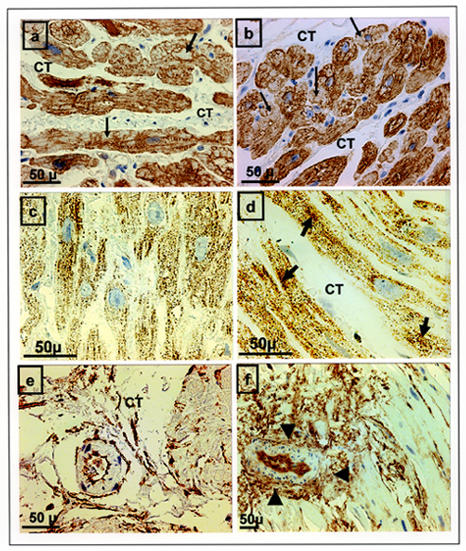
Immunohistochemical staining for desmin (**a,b**), mitochondria (**c,d**) and CD 34 (**e,f**) comparing before (**a,c,e**) and after (**b,d,f**) microwave ablation. Tissues after ablation showed more clear foci of coagulative necrosis with accumulation of mitochondria (thin arrows), and myolysis areas replacing the myofibrillar elements (thick arrows). The majority of the small intramyocardial vessels staining with CD 34 antibodies showed distinct occlusion of their lumens postablation (arrowheads in **f**). Panels **a, b** and **e** are at the same magnification. Panels **c** and **d** are at the same magnification.

Using immunohistochemical staining procedures, we assessed the expression and organization of contractile and cytoskeletal proteins of myocytes and extracellular matrix damaged before and after of microwave ablation procedure [24]. The acute changes of the atrial myocytes in endocardial post-ablation samples showed severe and complete loss of regularly arranged contractile material with an irregular proliferation or accumulation of one or more cytoplasmic components such as mitochondria (which were unusually small with the occasional giant mitochondria) and glycogen granules replacing the myofibrillar elements (Figure 2). The majority of the small intramyocardial vessels staining with CD34 and CD31 antibodies within the zone of the ablation lesion showed distinct occlusion of their lumens and severe disruption of endothelial or adventitial layers (Figure 2).

**Figure 3:**
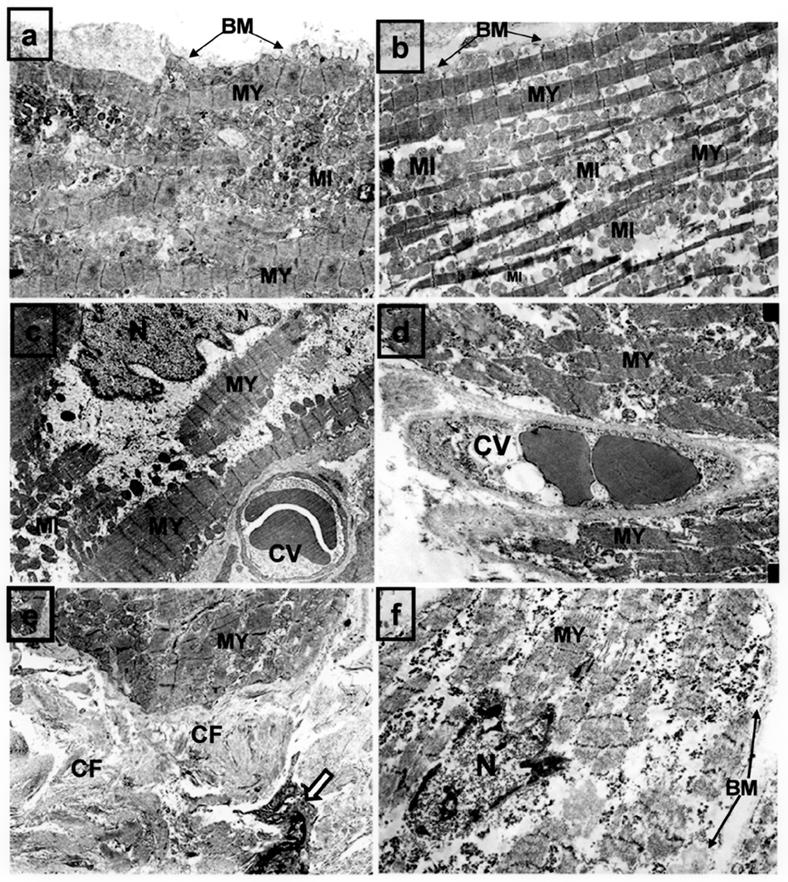
Transmission electron photomicrograph of samples preablation (**a,c,e**) and postablation using microwave (**b,d,f**) showing the architecture of contractile elements and basement membrane (**a,b**). The capillary vessel (CV) in (d) shows severe disruption of endothelial and adventitial layers compared with the vessel in preablation sample(**c**). In panel (**e**) the extracellular matrix show in preablation an collagen fibers with a fibroblast(open arrow) in the same field. A severely degenerated myocyte in postablation sample shown in (**f**) has severe disruption of nuclear and basement membranes, disarray and loss of contractile filaments and condensation of heterochromatin. Magnification: (**a**)x6000 (**b**)x4800 (**c**)x3790 (**d**)5910 (**e**)x5800 (**f**)x7540 BM=basement membrane; CF=collagen fibers; MI=mitochondria; MY=myocyte; N=nucleus.

Ultrastructural observations obtained before microwave ablation showed only focal alterations such as focal loss of contractile elements, abnormally shaped mitochondria, large and highly lobulated nuclei, intercalated disk disruption and basement membrane disruption (Figure 3). However, acute ultrastructural changes in myocyte endocardial post-ablation samples demonstrated irregular or complete loss of membranous borders, loss or very irregular shape of nuclei, mitochondrial swelling, disruption of endothelial cells of the capillary vessels, and signs of inflammation shown by the presence of macrophages [24] (Figure 3). These are morphologic features of irreversible injury indicative of non viable cells. Nonetheless, severely degenerated myocytes were isolated from other cells by fibrous tissue but occasionally were connected by junctional areas to only moderately degenerated cells. It is unclear whether these myocytes would remain viable over time to be capable of delivering electrical activity associated with AF. For these reasons, it is necessary to explore short- and long-term histologic changes following microwave energy ablation to evaluate it the full thickness lesion is a prerequisite for a lesion set to be effective.

Watanabe et al [25]. observed 2 days after application of epicardial microwave energy at the free wall of the left ventricle of mongrel dogs necrotic muscle with intramural hemorrhage that was demarcated from the normal myocardium by young granulation tissue on the border zone of ablated lesion. At 1 month, the necrosis was present only in the center of the lesion, and most of the necrotic muscle was replaced by fibrotic tissue. Similar results were found by Van Brakel et al [29] (Table 1). Healing by fibrosis was complete by 3 months and fatty tissue infiltrated a small area of the ablated lesion. After 6 months, the necrotic myocardium healed to a white-colored hard scar tissue which was sharply demarcated from normal myocardium. At 12 months the scar tissue consisted of collagen fibres and was infiltrated by fatty tissue [25]. However, little information is available on the thinner atrial wall regarding the histologic changes long-term after microwave ablation.

In summary, it seems that microwave ablation creates transmural lesions with only a few proarrhythmic events occurring during ablation, and suggests that application of this energy can be particularly useful in the treatment of tachyarrhythmias arising from deep foci of ventricular myocardium. Further studies are required to assess the use of this energy on atrial wall that is irregular in thickness [35][36].

## Figures and Tables

**Table 1:**
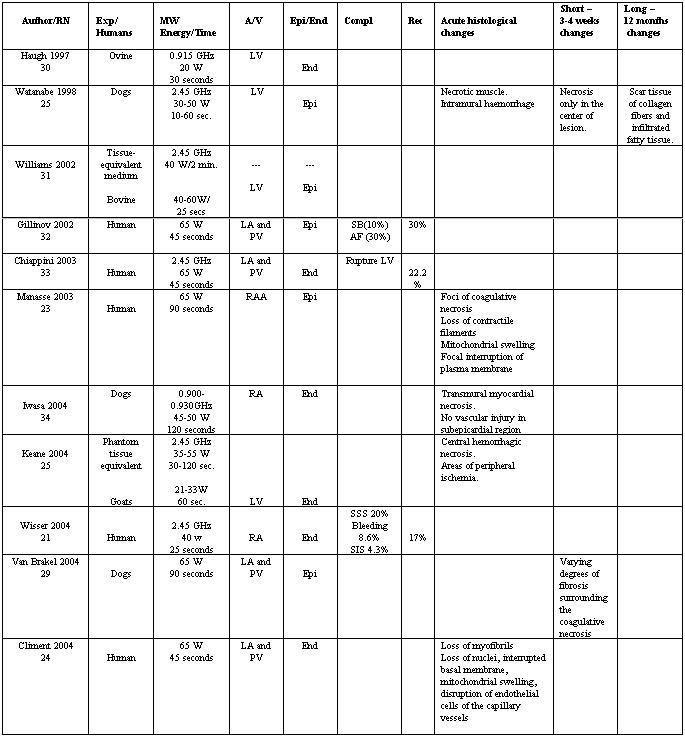


RN= Reference number. Exp: Experimental animal. A/V: Atrial/Ventricle. LA: Left Atrium. RA: Right Atrium. RA: Right Atrial Appendage. PV: Pulmonary Veins. LV: Left ventricle Epi/End: Epicardium/Endocardium Comp: Complications. Rec: Recurrences SSS: Sick Sinus Syndrome. SB: Sinus Bradycardia. AF= Atrial fibrillation SIS: Systemic Inflammatory Syndrome
